# A Dynamic Contrast‐Enhanced MRI‐Based Vision Transformer Model for Distinguishing HER2‐Zero, ‐Low, and ‐Positive Expression in Breast Cancer and Exploring Model Interpretability

**DOI:** 10.1002/advs.202503925

**Published:** 2025-06-09

**Authors:** Xu Zhang, Yi‐Yuan Shen, Guan‐Hua Su, Yuan Guo, Ren‐Cheng Zheng, Si‐Yao Du, Si‐Yi Chen, Yi Xiao, Zhi‐Ming Shao, Li‐Na Zhang, He Wang, Yi‐Zhou Jiang, Ya‐Jia Gu, Chao You

**Affiliations:** ^1^ Department of Radiology Fudan University Shanghai Cancer Center Fudan University Shanghai 200032 China; ^2^ Key Laboratory of Breast Cancer Department of Breast Surgery Fudan University Shanghai Cancer Center Fudan University Shanghai 200032 China; ^3^ Department of Oncology Shanghai Medical College Fudan University Shanghai 200032 China; ^4^ Institute of Science and Technology for Brain‐inspired Intelligence Fudan University Shanghai 201203 China; ^5^ Department of Radiology The First Hospital of China Medical University Shenyang 110001 China; ^6^ Department of Radiology Guangzhou First People's Hospital Guangzhou 510180 China

**Keywords:** breast cancer, deep learning, medical imaging, transcriptomics analysis

## Abstract

Novel antibody‐drug conjugates highlight the benefits for breast cancer patients with low human epidermal growth factor receptor 2 (HER2) expression. This study aims to develop and validate a Vision Transformer (ViT) model based on dynamic contrast‐enhanced MRI (DCE‐MRI) to classify HER2‐zero, ‐low, and ‐positive breast cancer patients and to explore its interpretability. The model is trained and validated on early enhancement MRI images from 708 patients in the FUSCC cohort and tested on 80 and 101 patients in the GFPH cohort and FHCMU cohort, respectively. The ViT model achieves AUCs of 0.80, 0.73, and 0.71 in distinguishing HER2‐zero from HER2‐low/positive tumors across the validation set of the FUSCC cohort and the two external cohorts. Furthermore, the model effectively classifies HER2‐low and HER2‐positive cases, with AUCs of 0.86, 0.80, and 0.79. Transcriptomics analysis identifies significant biological differences between HER2‐low and HER2‐positive patients, particularly in immune‐related pathways, suggesting potential therapeutic targets. Additionally, Cox regression analysis demonstrates that the prediction score is an independent prognostic factor for overall survival (HR, 2.52; *p* = 0.007). These findings provide a non‐invasive approach for accurately predicting HER2 expression, enabling more precise patient stratification to guide personalized treatment strategies. Further prospective studies are warranted to validate its clinical utility.

## Graphical abstract

This study develops a Vision Transformer‐based DCE‐MRI model for the non‐invasive classification of HER2 expression in breast cancer, demonstrating robust performance across multicenter cohorts. By integrating transcriptomic analysis, the model reveals immune‐related pathway differences among distinct HER2 expression levels. This approach offers insights into treatment stratification and advances precision oncology.

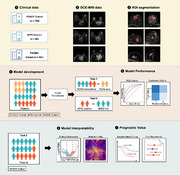

## 1 Introduction

Breast cancer is the most common malignant tumor among women worldwide and one of the leading causes of cancer‐related deaths.^[^ ^1^ ^]^ Human epidermal growth factor receptor 2 (HER2) plays a critical role in the molecular subtyping and treatment of breast cancer, with its expression status directly affecting patient prognosis and therapeutic strategies.^[^ ^2^ ^]^ With the emergence of novel antibody‐drug conjugates (ADCs), the subtype of HER2‐low expression (immunohistochemistry [IHC] 1+ or IHC 2+ with in situ hybridization [ISH] ‐) has gradually gained attention. Precisely distinguishing between HER2‐zero, HER2‐low, and HER2‐positive is crucial for identifying potential beneficiaries of ADCs therapies.^[^ ^3^, ^4^ ^]^ Recent clinical trials have demonstrated that patients with HER2‐low breast cancer significantly benefit from novel HER2‐targeted ADCs.^[^ ^3^, ^5^ ^]^ Unlike HER2‐positive patients who traditionally respond to HER2‐targeted therapies, HER2‐low patients were previously classified as HER2‐negative and lacked effective targeted treatment options. Accurate differentiation among HER2‐zero, HER2‐low, and HER2‐positive tumors is therefore essential, as misclassification may result in missed opportunities for appropriate ADCs therapies and suboptimal clinical outcomes. Traditional pathological techniques rely on invasive tissue sampling and may not fully capture the tumor heterogeneity. Therefore, developing a non‐invasive and reliable method to preoperatively assess HER2 expression status in breast cancer is essential for optimizing treatment strategies.

Magnetic resonance imaging (MRI) plays a significant role in the diagnosis, staging, and therapeutic monitoring of breast cancer.^[^ ^6^, ^7^, ^8^, ^9^ ^]^ MRI‐based radiomics can extract high‐throughput quantitative features from medical images, reflecting tumor heterogeneity and microenvironment characteristics.^[^ ^8^, ^9^ ^]^ However, traditional radiomics methods require manual feature extraction and have limited interpretability, making it difficult to fully elucidate the biological mechanisms behind model decisions.

Vision Transformer (ViT), a deep learning architecture that utilizes self‐attention mechanisms to capture both global and local information in images,^[^ ^10^, ^11^ ^]^ has achieved significant results in medical image analysis. However, the black‐box nature of deep learning models limits insight into their decision‐making processes, posing challenges for clinical adoption. Radiogenomics, by integrating imaging features with multi‐omics data, provides biological interpretability for the model.^[^ ^12^ ^]^ Studies have shown that the predictive results of deep learning models are correlated with tumor gene expression patterns, pathway activities, and tumor microenvironment features.^[^ ^13^, ^14^, ^15^, ^16^ ^]^ By conducting bioinformatics analysis on the model's predictions, it is possible to explore the relationship between model decisions and tumor biological characteristics, revealing the biological interpretability of the models and promoting their application in clinical practice.

In this study, we developed a ViT deep learning model using DCE‐MRI data to non‐invasively distinguish HER2 expression in breast cancer. Next, we evaluated the interpretability of the model's decisions using attention maps and transcriptomics correlation analysis. Furthermore, given the prognostic value of HER2 status, we hypothesized that the features the model focuses on during decision‐making may correlate with tumor biological characteristics and prognosis and the prediction score is a significant independent predictor of overall survival (OS).

## 2 Experimental Section

### 2.1 Ethics Statement

The multicenter retrospective study was conducted in accordance with the Declaration of Helsinki. Ethics approval was obtained from the Institutional Review Board of Fudan University Shanghai Cancer Center (NCT04461990), as well as from the ethics boards of Guangzhou First People's Hospital and the First Affiliated Hospital of China Medical University. All participants provided written consent after being informed.

### 2.2 Study Patients

We retrospectively collected three independent patient cohorts. The Fudan University Shanghai Cancer Center (FUSCC) cohort included 879 breast cancer patients treated at Fudan University Shanghai Cancer Center between November 2011 and January 2016. The Guangzhou First People's Hospital (GFPH) cohort included 100 patients treated at Guangzhou First People's Hospital between November 2017 and December 2022. The First Hospital of China Medical University (FHCMU) cohort included 140 patients treated at the First Hospital of China Medical University between October 2018 and December 2021. The inclusion criteria were as follows: a) pathologically confirmed primary breast cancer; b) availability of pretreatment MRI images; and c) HER2 expression status determined by the IHC and/or ISH of postsurgical specimens. The exclusion criteria included a) incomplete DCE‐MRI; b) severe motion artifacts; and c) multiple lesions with different pathological HER2 expression status. Finally, 708 samples were retained in the FUSCC cohort, 80 in the GFPH cohort, and 101 in the FHCMU cohort. The clinical characteristics of all three cohorts are summarized in **Table** **1**. In addition, the FUSCC cohort included follow‐up data of each patient (*n* = 708), among whom 367 had matched RNA sequencing data obtained from surgically resected tumor specimens, allowing for integrated analysis of imaging and transcriptomic profiles.

**Table 1:** Summary of Demographic and Clinical Data from Three Study Cohorts.

Variable	FUSCC cohort	GFPH cohort	FHCMU cohort
	(*n* = 708)	(*n* = 80)	(*n* = 101)
Age (y)^a)^	52.6 (10.5)	54.4 (9.3)	49.2 (10.4)
Menopause			
YES	403 (56.9)	56 (70.0)	44 (43.6)
NO	301 (42.5)	24 (30.0)	57 (56.4)
NA	4 (5.6)	0	0
Histology			
Invasive	648 (91.5)	73 (91.3)	93 (92.1)
Others	60 (8.5)	7 (8.7)	8 (7.9)
pT stage			
T1	303 (42.8)	15 (18.8)	6 (5.9)
T2	379 (53.5)	15 (18.8)	64 (63.4)
T3	19 (2.7)	1 (1.3)	21 (20.8)
T4	0	0	10 (9.9)
NA	7 (1.0)	49 (61.1)	0
pN stage			
N0	365 (51.6)	23 (28.6)	17 (16.8)
N1	182 (25.7)	6 (7.5)	57 (56.4)
N2	89 (12.6)	1 (1.3)	15 (14.9)
N3	66 (9.3)	1 (1.3)	12 (11.9)
NA	6 (0.8)	49 (61.1)	0
Grade			
G1	5 (0.7)	8 (10.0)	0
G2	272 (38.4)	35 (43.7)	73 (72.3)
G3	369 (52.1)	12 (15.0)	27 (26.7)
NA	62 (8.8)	25 (31.3)	1 (1.0)
ER status			
Positive	401 (56.6)	57 (71.2)	64 (63.4)
Negative	307 (43.4)	23 (28.8)	37 (36.6)
PR status			
Positive	343 (48.4)	51 (61.7)	66 (65.3)
Negative	365 (51.6)	29 (36.3)	35 (34.7)
HER2 status			
Positive	226 (31.9)	38 (47.5)	22 (21.8)
Low	344 (48.6)	32 (40.0)	53 (52.5)
Zero	138 (19.5)	10 (12.5)	26 (25.7)
Recurrence free survival (m)^b)^			
	78.1 (37.6‐90.6)	NA	NA
Overall survival (m)^b)^			
	80.4 (52.4–91.3)	NA	NA

Note—Except where indicated, data are numbers of women with percentages in parentheses. HER2 = human epidermal growth factor receptor 2; ER = estrogen receptor; PR = progesterone receptor; NA = not available;

^a)^ Data are means ± SDs;

^b)^ Data are medians, with IQRs in parentheses.

**Figure 1:**
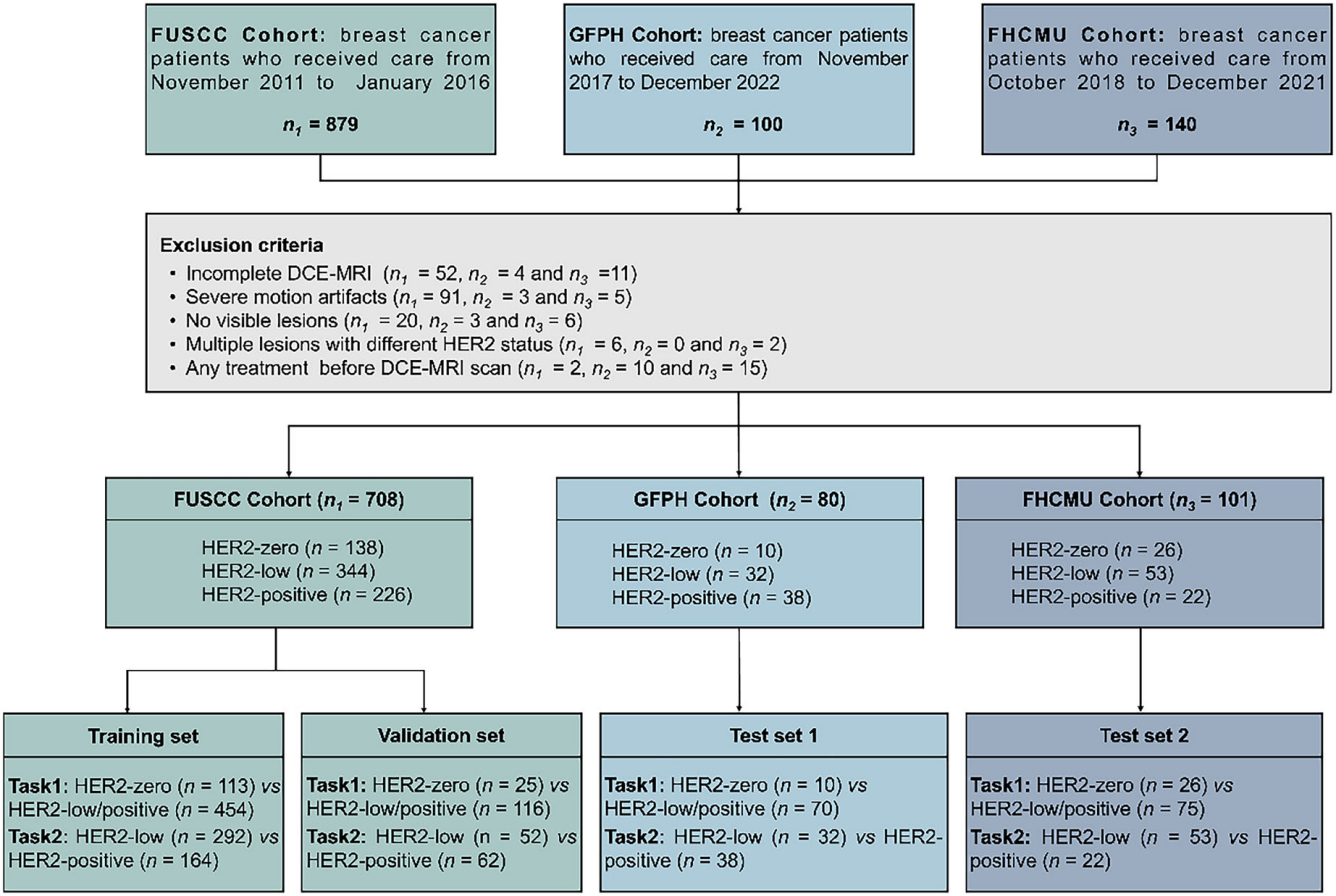
Data curation flowchart. DCE‐MRI = dynamic contrast‐enhanced magnetic resonance imaging. HER2 = human epidermal growth factor receptor 2.

Second, a diagnostic framework comprising two classification tasks was designed. Task 1 aimed to distinguish HER2‐zero from HER2‐low/positive breast cancers, given that patients with HER2‐zero tumors have not demonstrated clinical benefit from currently approved HER2‐targeted ADCs. Task 2 aimed to distinguish HER2‐low from HER2‐positive breast cancers, with the goal of refining treatment decision‐making among potential candidates for novel ADCs therapies. For both tasks, patients from the FUSCC cohort were randomly allocated into training and validation sets at a ratio of 8:2, and patients from the GFPH cohort and FHCMU cohort were used as external test sets. The study flowchart is shown in **Figure** **1**. Detailed patient distributions for training, validation, and test sets are provided in Supporting Information.

### 2.3 HER2 Expression Status Classification

HER2 expression status was determined by postsurgical specimens following the American Society of Clinical Oncology/College of American Pathologists guidelines.^[^ ^17^ ^]^ HER2‐zero was defined as an IHC score of 0, HER2‐low as an IHC score of 1+ or 2+ with a negative ISH result, and HER2‐positive as an IHC score of 3+ or 2+ with a positive ISH result.

### 2.4 Clinical Outcomes

For prognosis prediction, the primary endpoint was relapse‐free survival (RFS), defined as the time from diagnosis to the first recurrence of locoregional recurrence, distant metastasis, a diagnosis of contralateral breast cancer, or death from any cause. The secondary endpoint was overall survival (OS), defined as the time from diagnosis to death from any cause. Patients without events were censored at the time of the last follow‐up.

### 2.5 Breast DCE‐MRI Acquisition and Evaluation

All preoperative examinations were performed on three MRI scanners. The DCE‐MRI data acquisition parameters and MRI protocols for each cohort are presented in Table S1 (Supporting Information) and Supporting Information.

**Figure 2:**
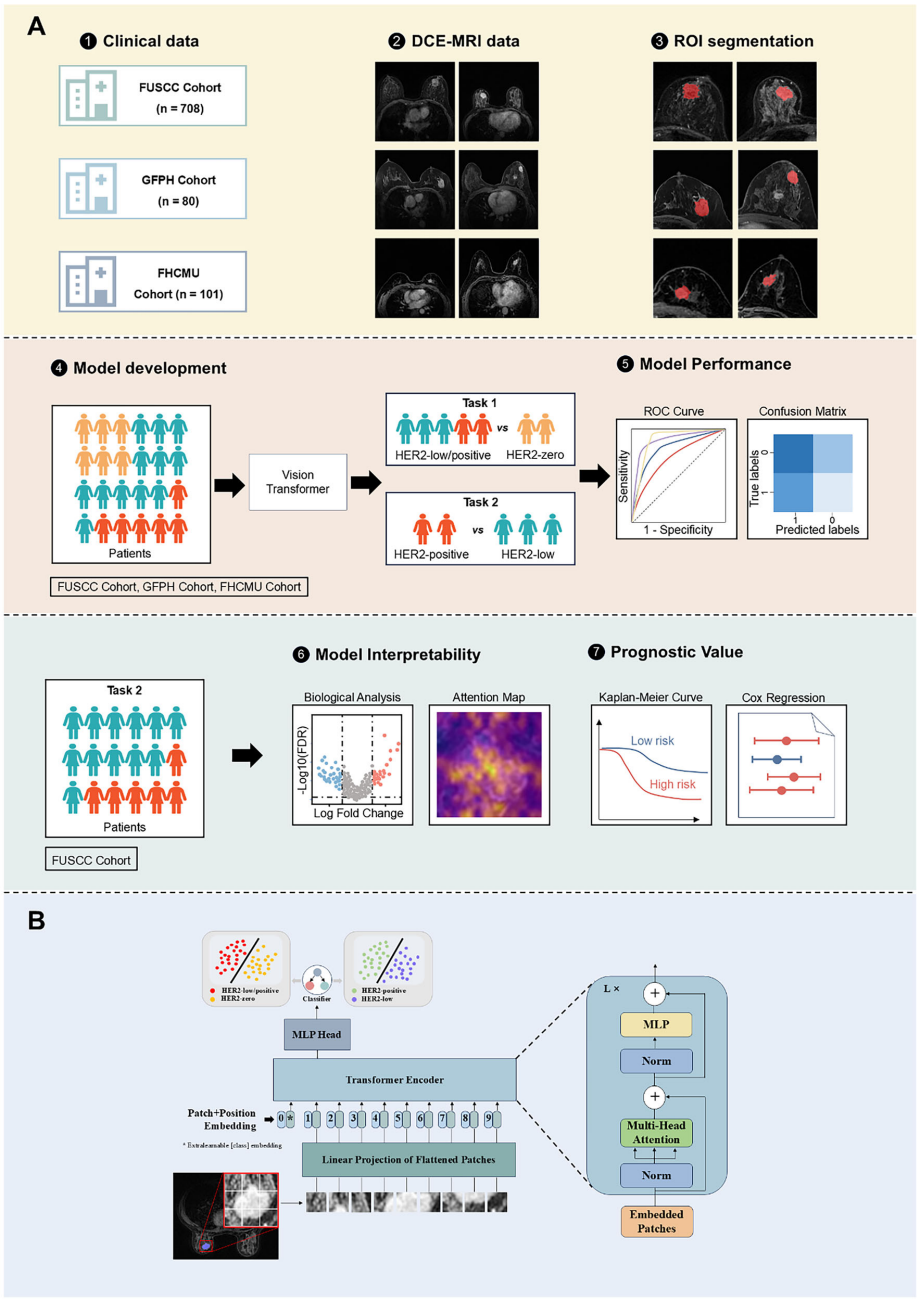
A) Overview of the workflow of this study. First, three independent breast cancer cohorts (FUSCC cohort: *n* = 708; GFPH cohort: *n* = 80; FHCMU cohort: *n* = 101) were integrated, and tumor regions of interest were manually segmented. Second, Vision Transformer (ViT) models were trained to perform two classification tasks: differentiating human epidermal growth factor receptor 2 (HER2)‐low/positive from HER2‐zero tumors, and then distinguishing HER2‐positive from HER2‐low tumors. Third, model performance was quantified using receiver operating characteristic (ROC) curves and confusion matrices. Fourth, for task 2 (distinguishing HER2‐positive from HER2‐low), interpretability was investigated using attention maps and transcriptomics analysis. Finally, Kaplan–Meier survival analysis and Cox proportional hazards regression were utilized for risk stratification to assess prognostic value. DCE = dynamic contrast‐enhanced. B) Detailed architecture of the Vision Transformer (ViT_tiny) model comprising 12 transformer layers, each with 3 parallel attention heads. Input images are processed with non‐overlapping 16 × 16 pixel patches, and the model operates with a hidden state dimension of 192. MLP = multilayer perceptron.

Tumor regions of interest (ROIs) were delineated semiautomatically on the early enhanced phase of DCE‐MRI by 3D Slicer software (version 4.10; www.slicer.org),^[^ ^18^ ^]^ with the largest cross‐sectional slice selected as the model input. ROI segmentation was performed by a radiologist (C.Y., with 9 years of experience in breast MRI), blinded to the histopathological results. To ensure reproducibility and the stability of segmentation outcomes, intraclass correlation coefficients (ICCs) were obtained from repeatability experiments, which involved tumor outlining and feature extraction. Sixty randomly selected samples from the FUSCC cohort were assessed for intra‐ and inter‐observer agreement. For intra‐observer evaluation, the same radiologist (C.Y.) repeated the ROI delineation for these cases after a two‐week interval. For inter‐observer evaluation, another radiologist (Y.Y.S., with 4 years of experience in breast MRI) independently performed ROI segmentation on the same cases, blinded to clinical and histopathological information. An ICC value greater than 0.75 was considered indicative of good agreement, and greater than 0.9 as excellent according to established guidelines. The concordance analysis revealed high consistency, with intra‐observer ICCs exceeding 0.9 and inter‐observer ICCs above 0.8, indicating reliable segmentation. Based on these results, the more experienced radiologist completed the entire ROI segmentation for each MRI scan layer. For the GFPH cohort and FHCMU cohort, tumor ROIs were manually delineated following the same protocol. The section containing the largest tumor region was chosen as the model's input (**Figure** **2**A).

### 2.6 Model Development

As shown in Figure 2, two ViT deep learning models were trained: 1) to distinguish HER2‐zero from HER2‐low and ‐positive breast cancer, and 2) to distinguish HER2‐low from HER2‐positive. Given the increasing clinical importance of HER2‐low in the context of novel ADCs, this study focused on distinguishing HER2‐low from HER2‐positive breast cancers to guide treatment strategies, following the initial exclusion of HER2‐zero tumors for whom current HER2‐targeted ADCs have not demonstrated clinical benefit. ViT leverages multi‐head self‐attention mechanisms to capture global relationships within images. The models were implemented using the ViT_tiny architecture from the timm library (https://github.com/huggingface/pytorch‐image‐models),^[^ ^19^ ^]^ with pretrained weights initialized from ImageNet. The models were trained on early‐phase enhanced tumor images from the DCE‐MRI training set and validated on the validation set and independent test sets. To further explore the interpretability of the model in distinguishing HER2‐low from HER2‐positive breast cancer, attention maps were generated to highlight regions critical for decision‐making,^[^ ^10^ ^]^ while transcriptomics analysis (including differential expression analysis and functional enrichment analysis^[^ ^20^, ^21^ ^]^) was conducted to investigate underlying biological mechanisms. Additional details on the ViT architecture, training parameters, and evaluation processes are provided in Supporting Information.

### 2.7 Transcriptomic Data Generation and Analysis

Sample processing for total RNA extraction, RNA sequencing procedures, and bioinformatic operations and analysis are presented in the Supporting Information.

### 2.8 Statistical Analysis

All statistical analyses were conducted using Python (version 3.8.0, https://www.python.org) and R software (version 4.1.2, https://www.r‐project.org). Patient characteristics were analyzed using the student's t‐test for continuous variables and the χ^2^ test or Fisher's exact test for categorical variables. The performance of the ViT models was evaluated using metrics including accuracy, sensitivity, specificity, and area under the receiver operating characteristic curve (AUC). The optimal classification thresholds were determined using the Youden index. Bootstrapped 95% confidence intervals (CI) were calculated using 1000 resamples. Hub genes were identified using the STRING^[^ ^22^ ^]^ (https://string‐db.org) database and Cytoscape^[^ ^23^ ^]^ (version 3.10.2, https://cytoscape.org). Survival curves for OS and RFS were estimated using the Kaplan‒Meier analysis, and the log‐rank test was used to compare survival between different groups. Hazard ratios (HR) with 95% CI were calculated using univariate and multivariate Cox proportional hazards regression models to identify prognostic factors associated with OS. All statistical tests were two‐sided, and a *p*‐value <05 was considered statistically significant.

## 3 Results

### 3.1 Clinical Characteristics

The clinical characteristics of patients in the FUSCC cohort (*n* = 708), GFPH cohort (*n* = 80), and FHCMU cohort (*n* = 101) are summarized in Table 1. The mean age was 52.6 years (SD, 10.5) for the FUSCC cohort, 54.4 years (SD, 9.3) for the GFPH cohort, and 49.2 years (SD, 10.4) for the FHCMU cohort. In the FUSCC cohort, the median RFS was 78.1 months (IQR, 37.6–90.6), and the median OS was 80.4 months (IQR, 52.4–91.3). For task 1 and task 2, no statistically significant differences in baseline characteristics were observed between the training and validation set (Tables S2 and S3, Supporting Information).

### 3.2 Performance of the Model in Distinguishing HER2‐Zero versus HER2‐Low and HER2‐Positive Status

**Figure 3:**
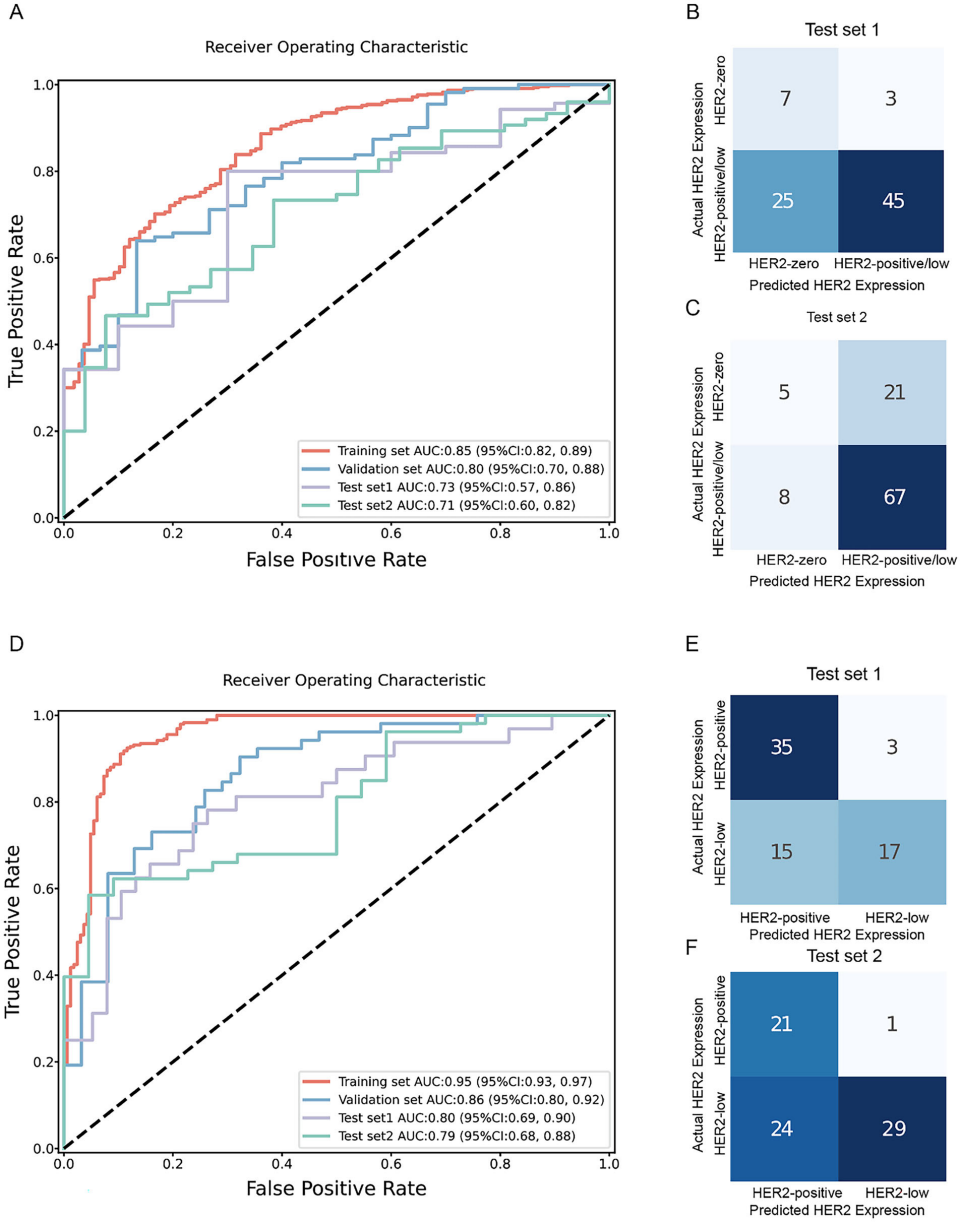
Performance of the Vision Transformer (ViT) model in distinguishing different status of human epidermal growth factor receptor 2 (HER2) expression. A) Receiver operating characteristic curves for distinguishing HER2‐low/positive from HER2‐zero, shown for the training, validation, and two independent test sets. B,C) Confusion matrices for the two independent test sets for the HER2‐low/positive versus HER2‐zero classification. D) Receiver operating characteristic curves for distinguishing HER2‐low from HER2‐positive, shown for the training, validation, and two independent test sets. E,F) Confusion matrices for the two independent test sets for the HER2‐low versus HER2‐positive classification. AUC = area under the receiver operating characteristic curve. CI = confidence intervals.

The performance of the model in distinguishing HER2‐zero from HER2‐low/positive across the training, validation, and test datasets is illustrated in **Figure** **3**A. The AUC was 0.85 for the training set, 0.80 for the validation set, 0.73 for the test set 1, and 0.71 for the test set 2.

The model achieved an accuracy, sensitivity, specificity, and F1 score of 0.73, 0.70, 0.83, and 0.81; in the training set; 0.74, 0.76, 0.67, and 0.82 in the validation set; 0.65, 0.64, 0.70 and 0.76 in the test set 1; and 0.71, 0.89, 0.19 and 0.82 in the test set 2, respectively (**Table** **2**, Figure 3B,C).

**Table 2:** Performance Metrics of the ViT Model for HER2 Classification Tasks.

Tasks and datasets	AUC [95% CI]	Accuracy [95% CI]	Sensitivity [95% CI]	Specificity [95% CI]	F1 score [95% CI]
Task 1					
Training set	0.85 (0.82, 0.89)	0.73 (0.69, 0.76)	0.70 (0.66, 0.74)	0.83 (0.75, 0.90)	0.81 (0.78, 0.83)
Validation set	0.80 (0.70, 0.88)	0.74 (0.66, 0.81)	0.76 (0.67, 0.83)	0.67 (0.50, 0.83)	0.82 (0.78, 0.87)
Test set 1	0.73 (0.57, 0.86)	0.65 (0.55, 0.75)	0.64 (0.53, 0.75)	0.70 (0.38, 1.00)	0.76 (0.71, 0.82)
Test set 2	0.71 (0.60, 0.82)	0.71 (0.62, 0.79)	0.89 (0.82, 0.96)	0.19 (0.05, 0.35)	0.82 (0.77, 0.87)
Task 2					
Training set	0.95 (0.93, 0.97)	0.91 (0.88, 0.94)	0.92 (0.89, 0.95)	0.88 (0.83, 0.93)	0.93 (0.91, 0.95)
Validation set	0.86 (0.80, 0.92)	0.77 (0.69, 0.84)	0.63 (0.51, 0.77)	0.89 (0.81, 0.95)	0.72 (0.65, 0.79)
Test set 1	0.80 (0.69, 0.90)	0.74 (0.63, 0.84)	0.53 (0.36, 0.71)	0.92 (0.82, 1.00)	0.65 (0.56, 0.75)
Test set 2	0.79 (0.68, 0.88)	0.67 (0.57, 0.76)	0.55 (0.42, 0.67)	0.95 (0.84, 1.00)	0.70 (0.64, 0.76)

Note.—Task 1 refers to the classification of HER2‐zero versus HER2‐low/positive breast cancer cases. Task 2 refers to the classification of HER2‐low versus HER2‐positive breast cancer cases. AUC = area under the receiver operating characteristic curve. CI = confidence interval.

### 3.3 Performance of the Model in Distinguishing HER2‐Low versus HER2‐Positive Status

The performance of the model in distinguishing HER2‐low from HER2‐positive across the training, validation, and test datasets is illustrated in Figure 3D. The AUC was 0.95 for the training set, 0.86 for the validation set, 0.80 for the test set 1, and 0.79 for the test set 2.

The model achieved an accuracy, sensitivity, specificity, and F1 score of 0.91, 0.92, 0.88, and 0.93 in the training set; 0.77, 0.63, 0.89, and 0.72 in the validation set; 0.74, 0.53, 0.92, and 0.65 in the test set 1; and 0.67, 0.55, 0.95, and 0.70 in the test set 2, respectively (Table 2, Figure 3E,F).

### 3.4 Attention Map Analysis of the Model for Distinguishing HER2‐Low and HER2‐Positive

**Figure 4:**
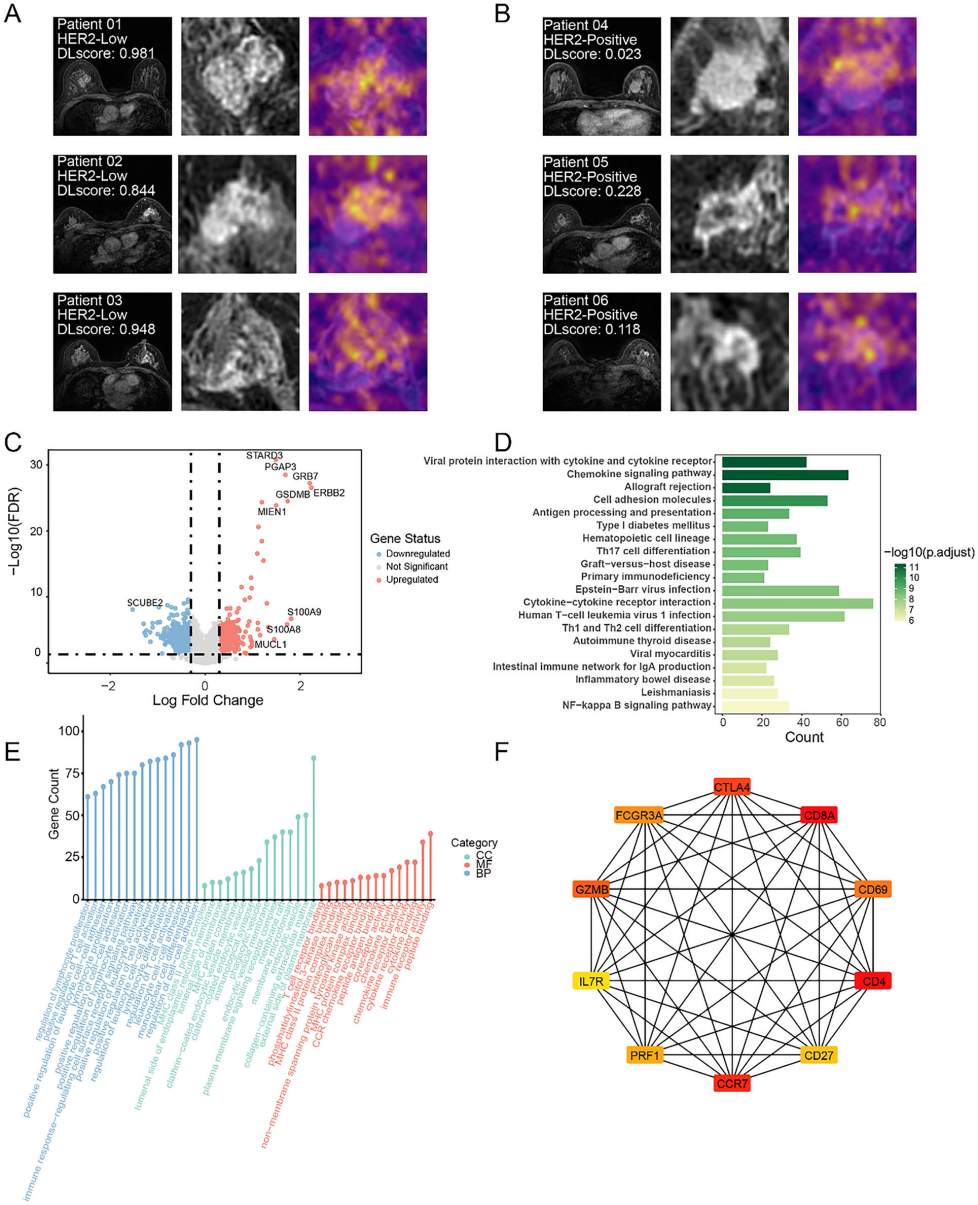
Interpretability of the Model for Distinguishing HER2‐Low and HER2‐Positive. Diagram presents attention maps for six representative patients, with three diagnosed as HER2‐Low A) and three diagnosed as HER2‐Positive B). In each panel, the first column shows the original MRI images with the deep learning (DL) score for each patient, the second column displays magnified regions of interest, and the third column overlays attention maps, highlighting areas that the model identified as being of high importance for its classification decision. C) Volcano plot showing the differentially expressed genes (DEGs) based on the predicted HER2‐Positive and HER2‐Low groups. Functional enrichment analysis of DEGs: D) GO enrichment analysis; E) KEGG enrichment analysis. F) Protein‐protein interaction (PPI) network of DEGs. Top 10 hub genes were identified using the Maximal Clique Centrality (MCC) method.

To improve the interpretability of our deep learning model in distinguishing HER2‐positive from HER2‐low breast cancer patients, attention maps were used to visualize pixel importance through color gradients, highlighting the most significant regions contributing to the model's decision. By visualizing pixel importance through color gradients, the maps highlighted distinct features in the imaging data. In the HER2‐low group, the attention maps exhibited broader and more intense activation regions reflecting the heterogeneous characteristics associated with HER2‐low tumors, while fewer and more focused regions were activated for the HER2‐positive group (**Figure** **4**A,B).

### 3.5 Biological Relevance of the Model for Distinguishing HER2‐Low and HER2‐Positive

To further investigate the biological relevance underlying our model's predictions in distinguishing HER2‐low from HER2‐positive breast cancer, we performed a differential gene expression analysis based on the model's predicted classifications. In total, 1173 differentially expressed genes (DEGs) were identified between the model‐predicted HER2‐low and HER2‐positive groups (Table S4, Supporting Information). Among these, *ERBB2*, *GRB7*, and *S100A9* were the most upregulated in HER2‐positive tumors, while *SCUBE2* was the most downregulated in HER2‐positive tumors. These genes are critical in tumor progression and HER2 signaling pathways.

Next, GO and KEGG analyses were performed to further elucidate the biological functions of the captured model‐based genes (Figure 4E,D; Figures S4 and S5; Table S5–S10, Supporting Information). The GO analysis revealed significant enrichment in immune‐related processes such as immune receptor activity (p.adjust <0.001) and cytokine receptor activity (p.adjust <0.001), which are central to immune modulation and tumor‐immune interactions. Additionally, pathways related to T cell receptor binding and MHC protein complex binding were also enriched (p.adjust <0.001), highlighting the involvement of antigen presentation and immune surveillance in shaping tumor‐immune dynamics (Figure 4E). The KEGG analysis supported these findings, with the top enriched pathways including cytokine‐cytokine receptor interaction (p.adjust <0.001) and chemokine signaling pathway (p.adjust <0.001). These pathways are central to immune system regulation and cell communication, further indicating the importance of immune signaling in the tumor microenvironment. Moreover, cell adhesion molecules (p.adjust <0.001) and antigen processing and presentation pathways were also enriched, suggesting that the model's predictions may capture biological differences in how immune cells engage with and recognize tumor cells (Figure 4D).

We then constructed a protein‐protein interaction (PPI) network using the DEGs and identified the top 10 hub genes through the Maximal Clique Centrality (MCC) method. These hub genes, including *CTLA4*, *CD4*, *CD8A*, *CCR7*, *CD27*, *GZMB*, *PRF1*, *IL7R*, *FCGR3A*, and *CD69*, are primarily involved in immune regulation and T‐cell activation, reflecting the model's focus on immune‐related mechanisms in distinguishing HER2‐positive from HER2‐low tumors. (Figure 4F).

### 3.6 Prognostic Value of the Model for Distinguishing HER2‐Low and HER2‐Positive

**Figure 5:**
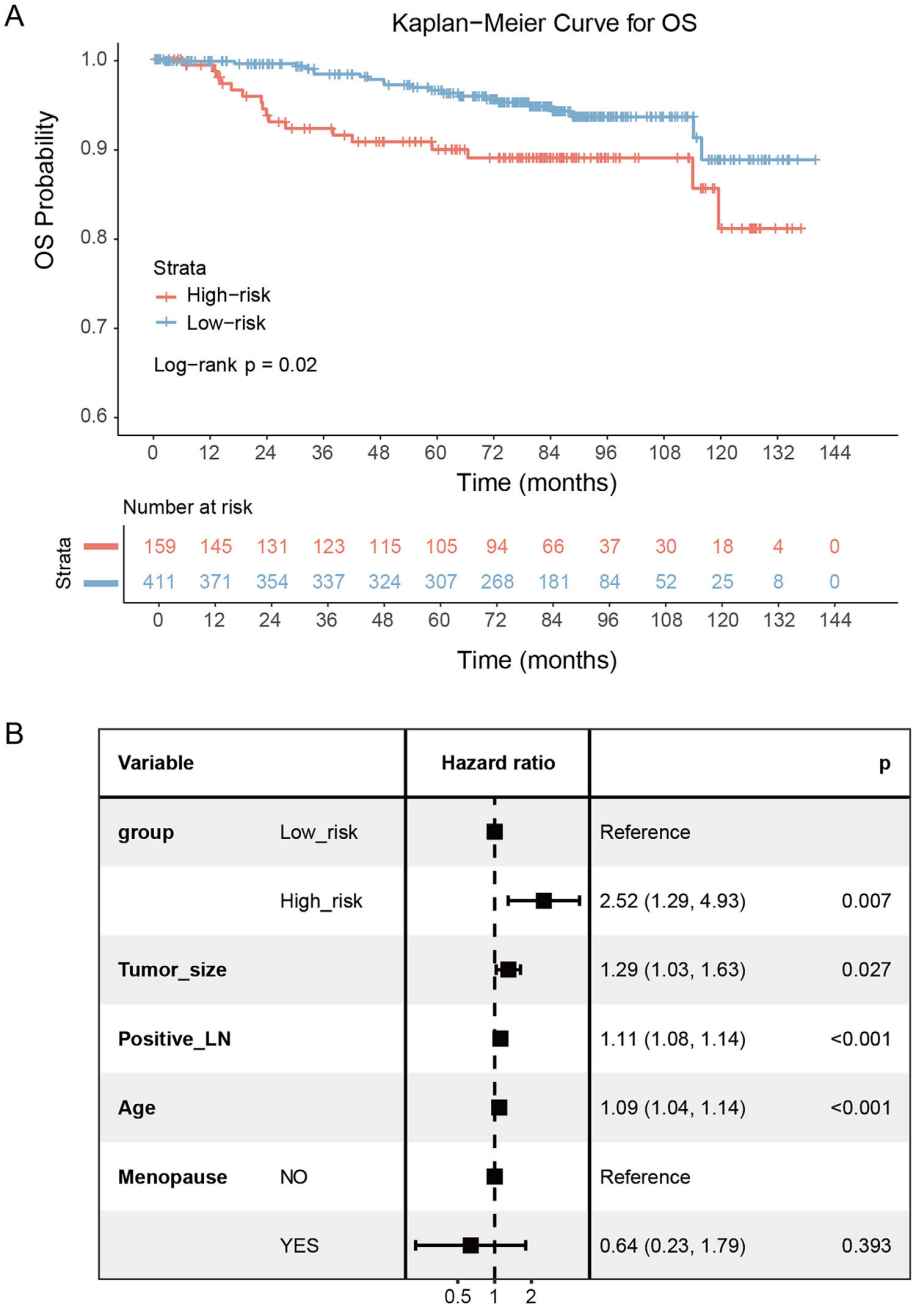
A) Kaplan–Meier analysis for overall survival (OS) based on high‐ and low‐risk groups (cutoff = 0.5) predicted by the Vision Transformer (ViT) model in distinguishing HER2‐low from HER2‐positive patients (*p* = 0.02 by log‐rank test) in FUSCC cohort. B) Forest plot of the multivariable Cox proportional hazards model based on OS in FUSCC cohort. LN = lymph node.

Moreover, to explore the prognostic value of the model, Kaplan‐Meier analysis was employed to evaluate RFS and OS based on both the actual HER2 status and the predicted risk groups (cutoff = 0.5, derived from model predictions) in the FUSCC cohort. Patients in the predicted high‐risk group had significantly poorer OS (*p* = 0.02, **Figure** **5**A), whereas no significant difference in OS was observed between groups defined by actual HER2 status (*p* = 0.069, Figure S1, Supporting Information). RFS showed no significant differences for either the predicted risk groups or the actual HER2 status group (both *p* > 0.05, Figures S2 and S3, Supporting Information). Univariable Cox proportional hazards analysis was conducted to assess the prognostic significance of the predicted risk group, tumor size, positive lymph nodes, age, menopausal status, and lymphovascular invasion status. Multivariable Cox analysis demonstrated the predicted high‐risk group as an independent OS prognostic factor (HR, 2.52; 95% CI, 1.29–4.93; p = 0.007; Figure 5B, **Table** **3**).

**Table 3:** Uni‐ and Multivariable Cox Regression Analyses for OS in FUSCC cohort.

Variable	Univariable HR	*p* value	Multivariable HR	*p* value
Group (high risk vs low risk)	2.10 (1.11, 3.99)	0.023	2.52 (1.29, 4.93)	0.007
Tumor size	1.33 (1.08, 1.62)	0.006	1.29 (1.03, 1.63)	0.027
Positive LN	1.12 (1.09, 1.14)	<0.001	1.11 (1.08, 1.14)	<0.001
Age	1.06 (1.02, 1.09)	<0.001	1.09 (1.04, 1.14)	<0.001
Menopause (yes vs no)	2.27 (1.10, 4.68)	0.026	0.64 (0.23, 1.79)	0.393
LVI (yes vs no)	1.92 (0.99, 3.72)	0.052		

Note.—Data in parentheses are 95% CIs. Group risk stratification was based on model predictions (cutoff = 0.5). LVI = lymphovascular invasion status. LN = lymph node.

## 4 Discussion

This study developed a ViT‐based deep learning model using DCE‐MRI to non‐invasively assess HER2 expression in breast cancer, identifying HER2‐low/positive cases and further distinguishing between HER2‐low and HER2‐positive subtypes. The model demonstrated robust performance across multicenter datasets, achieving AUC values of 0.73 and 0.71 in external test sets for distinguishing HER2‐zero from HER2‐low/positive cancers, and 0.80 and 0.79 for distinguishing HER2‐low from HER2‐positive cancers. Transcriptomic analysis revealed immune microenvironment features specific to HER2‐low tumors, supporting the model's biological interpretability. Moreover, survival analysis showed the model's prediction scores, derived from distinguishing HER2‐low from HER2‐positive patients, as independent predictors of overall survival (OS), highlighting the clinical value of this model.

Accurate distinguishing HER2‐low breast cancers is particularly critical, as HER2‐low patients have now emerged as a distinct therapeutic subgroup eligible for novel ADCs treatments.^[^ ^24^ ^]^ HER2‐zero patients generally do not benefit from HER2‐targeted therapies. Our ViT model addresses this clinical need, supporting more precise therapeutic decision‐making and potentially expanding the pool of patients who can benefit from targeted therapies. Previous studies have utilized multiparametric MRI, incorporating sequences such as DWI, T2WI, and DCE‐MRI to explore MRI‐based radiomics for HER2 characterization.^[^ ^25^, ^26^, ^27^, ^28^, ^29^ ^]^ Ramtohul et al. developed MRI radiomics models using multiparametric MRI sequences to differentiate HER2‐zero from HER2‐low/positive cancers, with an AUC of 0.80 in the external test set.^[^ ^25^ ^]^ Similarly, Chen et al. employed habitat imaging based on multiparametric MRI to quantify intratumoral heterogeneity, demonstrating remarkable performance in differentiating HER2‐positive, HER2‐low, and HER2‐zero breast cancers.^[^ ^28^ ^]^

Building on these findings, our study utilized DCE‐MRI‐based ViT models and achieved robust and substantial performance, especially in distinguishing HER2‐low from HER2‐positive breast cancers, likely due to the ViT's advanced feature extraction capabilities, which leverage self‐attention mechanisms to capture both global and local imaging patterns. Compared with convolutional neural networks (CNNs), which rely on local receptive fields and hierarchical feature aggregation, ViT models employ self‐attention mechanisms that enable direct modeling of long‐range dependencies and global contextual relationships within medical images. This architecture allows ViT to better preserve spatial coherence and capture complex feature interactions, particularly important in cases like HER2‐low breast cancer where subtle heterogeneity can influence clinical decision‐making. However, relying on a single sequence may limit the model's ability to capture the additional discriminative information provided by other MRI sequences. Meanwhile, the biological similarities between HER2‐zero and HER2‐low tumors, both traditionally classified as HER2‐negative, lead to overlapping imaging features that further complicate differentiation. Together, these factors likely contribute to the slightly lower AUCs observed when distinguishing HER2‐zero from HER2‐low/positive cancers.

Additionally, the modeling approaches employed in previous studies have predominantly been based on traditional radiomics, which involves manual feature extraction and selection, potentially missing intricate spatial relationships within imaging data, while studies leveraging deep learning remain relatively limited.^[^ ^30^, ^31^ ^]^ Guo et al further advanced this field by integrating radiomics with deep learning features to develop a deep learning radiomics (DLR) model, which outperformed traditional radiomics models in distinguishing HER2 status.^[^ ^30^ ^]^ This approach highlighted the benefits of combining manual radiomic features with automatically learned deep features to enhance model performance. In contrast, our ViT‐based deep learning model learns and integrates both global and local imaging features through self‐attention mechanisms in an end‐to‐end manner. This not only enhances the model's ability to detect nuanced differences in HER2 expression but also improves robustness across multicenter datasets. By eliminating the need for manual feature extraction and leveraging the powerful feature representation capabilities of ViT, our model effectively captures the subtle imaging patterns associated with different HER2 statuses. Given the advantages the model, it has the potential to bring potential value to clinical workflows. On the one hand, it enables non‐invasive HER2 status evaluation based on DCE‐MRI, potentially serving as a “virtual biopsy” to reduce the invasiveness and associated risks of traditional tissue biopsies. On the other hand, it facilitates dynamic monitoring of HER2 status during treatment, allowing clinicians to adjust therapeutic strategies in a timely manner.

To elucidate the biological relevance of our ViT model, we integrated attention map analysis with gene expression profiling to investigate the molecular underpinnings of HER2 status differentiation. Our findings demonstrated that the broader regions of activation identified in HER2‐low tumors align with the known heterogeneity of this subtype.^[^ ^32^, ^33^ ^]^ The differential expression of key genes further underscores the biological divergence between HER2‐low and HER2‐positive tumors, particularly involving tumor proliferation, HER2 signaling activation, and immune regulation. *ERBB2*, encoding the HER2 receptor, was markedly upregulated in HER2‐positive tumors, supporting its central role in oncogenic pathway activation and tumor proliferation.^[^ ^34^ ^]^ *GRB7*, frequently co‐amplified with ERBB2, enhances tumor invasion and metastatic potential, contributing to the more aggressive phenotype observed in HER2‐positive cancers.^[^ ^35^ ^]^ *S100A9*, a calcium‐binding protein, is upregulated in HER2‐positive tumors and modulates the tumor immune microenvironment by promoting metabolic reprogramming and reducing lymphocyte infiltration.^[^ ^36^, ^37^ ^]^ Although direct comparisons between HER2‐low and HER2‐positive tumors are limited, the known functions of *S100A9* suggest its involvement in shaping distinct immune dynamics associated with HER2 status. In contrast, *SCUBE2*, a protein with context‐dependent tumor suppressor functions, was found upregulated in HER2‐positive tumors in our analysis. This observation is consistent with previous studies indicating that *SCUBE2* expression is associated with chemotherapy resistance and may influence clinical outcomes in breast cancer^[^ ^38^, ^39^ ^]^ Pathway enrichment analysis further revealed that these gene expression differences were associated with immune‐related biological processes, including immune response regulation, antigen presentation, and cell adhesion. These pathways are critical for modulating tumor‐immune dynamics and suggest unique biological interactions within HER2‐low tumors. This interpretability bridges the gap between imaging‐based predictions and underlying tumor biology, offering a deeper understanding of the mechanisms driving HER2‐low status. Moreover, the model's ability to noninvasively characterize molecular features highlights its clinical potential for stratifying patients who may benefit from HER2‐targeted therapies, including antibody‐drug conjugates (ADCs), which have shown increasing efficacy in HER2‐low cases, emphasizing the need for precise subgroup identification to optimize treatment outcomes.

Despite its strengths, our study acknowledges several limitations in the imaging‐based prediction of HER2‐low status. First, the biological similarity between HER2‐low and HER2‐zero tumors complicates precise differentiation based solely on imaging features. Second, while attention maps and gene expression correlations have enhanced the interpretability of our deep learning model, the inherent black‐box nature of such models limits a comprehensive understanding of how specific imaging features drive predictions. Future research could address this by integrating deep learning with habitat imaging, enabling more granular exploration of tumor microenvironment heterogeneity and improving model interpretability. Finally, the retrospective design and variability in imaging protocols across centers may limit the model's generalizability, underscoring the need for prospective studies with standardized protocols and larger, diverse cohorts to validate its clinical applicability and enhance predictive accuracy.

In conclusion, our ViT‐based model offers a novel and effective approach for non‐invasive HER2 status assessment in breast cancer, bridging imaging‐derived features with molecular biology to provide clinically relevant insights. By capturing tumor heterogeneity and its associated molecular pathways, such as immune response and antigen presentation, the model not only enhances biological interpretability but also supports the identification of patients who may benefit from emerging HER2‐targeted therapies, including antibody‐drug conjugates. Furthermore, the observed correlation between model predictions and survival outcomes highlights its potential utility in prognostic evaluation and treatment stratification. Compared to traditional methods like IHC and ISH, this MRI‐based model represents a comprehensive and non‐invasive alternative, addressing challenges related to sampling bias and procedural invasiveness. These findings underscore the model's promise in advancing precision oncology and improving outcomes for breast cancer patients through tailored therapeutic strategies.

## Conflict of Interest

The authors declare no conflict of interest.

## Author Contributions

X.Z., Y.Y.S., G.H.S., and Y.G. contributed equally to this work and shared the co‐first authors. Y.J.G. and C.Y. conceptualized the study; X.Z. and Y.Y.S. developed the methodology. Y.Y.S., R.C.Z., S.Y.D., S.Y.C., Z.M.S., and Y.G. curated the data. G.H.S. and Y.X. conducted the investigation. G.H.S. performed the formal analysis. X.Z. provided statistical guidance. X.Z. and Y.Y.S. drafted the original manuscript. Y.J.G. and C.Y. reviewed and edited the manuscript. Y.J.G., L.N.Z., Y.Z.J., H.W., and C.Y. supervised the project. All authors approved the final manuscript, and the corresponding author, C.Y., affirmed that all listed authors meet the authorship criteria.

## Supporting information



Supporting Information

Supplemental Tables

## Data Availability

All data in this study are available from the corresponding author by reasonable request. All codes used for models and data analysis are available on GitHub (https://github.com/2023XuZhang9/DL‐VIT‐HER2).
