# Supplementary material for: A Dynamic Contrast‐Enhanced MRI‐Based Vision Transformer Model for Distinguishing HER2‐Zero, ‐Low, and ‐Positive Expression in Breast Cancer and Exploring Model Interpretability
Source: Adv Sci (Weinh). 2025 Jun 9;12(33):e03925. doi: 10.1002/advs.202503925 (PMC12412478; doi:10.1002/advs.202503925)
Supplement: Supplementary file 1 — Supporting Information [file ADVS-12-e03925-s001.docx]

A Dynamic Contrast-Enhanced MRI-Based Vision Transformer Model for Distinguishing HER2-Zero, -Low, and -Positive Expression in Breast Cancer and Exploring Model Interpretability

Supporting Information

Detailed Patient Distribution for Distinguishing HER2-Low/Positive from HER2-Zero and Distinguishing HER2-Low from HER2-Positive breast cancers

For task 1, 708 breast cancer patients from FUSCC cohort were randomly allocated into the training set (n = 567, included 454 HER2-low/positive and 113 HER2-zero expression) and validation set (n = 141, included 116 HER2-low/positive and 25 HER2-zero expression) at a ratio of 8:2. Additionally, breast cancer patients from GFPH cohort and FHCMU cohort were used as test set 1 (n = 80, included 70 HER2-low/positive and 10 HER2-zero expression) and test set 2 (n = 101, included 75 HER2-low/positive and 26 HER2-zero expression), respectively.

For task 2, 570 HER2-low/positive breast cancer patients from FUSCC cohort were randomly allocated into the training set (n = 456, included 292 HER2-low and 164 HER2-positive expression) and validation set (n = 114, included 52 HER2-low and 62 HER2-positive expression) at a ratio of 8:2. Additionally, HER2-low/positive breast cancer patients from GFPH cohort and FHCMU cohort were used as test set 1 (n = 70, included 32 HER2-low and 38 HER2-positive expression) and test set 2 (n = 75, included 53 HER2-low and 22 HER2-positive expression), respectively.

DCE-MRI protocols

For FUSCC cohort, all patients underwent preoperative breast DCE-MRI examination using a 1.5 T Aurora system with specialized breast coils. The imaging was performed using a T1-weighted sequence. The imaging parameters included: time of repetition (TR) = 29 msec, time of echo (TE) = 4.8 msec, field of view (FOV) = 360 mm × 360 mm, slice thickness = 1.5 mm, slice gap = 1.5 mm, flip angle = 15°, matrix of 360 × 360, and number of slices = 108. The contrast medium Gd-DTPA (0.1 mmol/kg, flow rate 2.0 mL/sec) was injected 90 seconds after the plain scan. Postcontrast images were obtained at 90, 180, and 360 seconds after injection.

For GFPH cohort, all patients underwent preoperative breast DCE-MRI examination using a 1.5 T United Imaging system with specialized breast coils. The imaging was performed using a T1-weighted sequence. The imaging parameters included: time of repetition (TR) = 5.1 msec, time of echo (TE) = 2.1 msec, field of view (FOV) = 360 mm × 360 mm, slice thickness = 1.2 mm, slice gap = 1.2 mm, matrix size of 416 × 416, and number of slices = 120. The contrast medium Gd-DTPA (0.1 mmol/kg, flow rate 2.0 mL/sec) was injected 90 seconds after the plain scan. Postcontrast images were obtained at 90, 180, and 360 seconds after injection.

For FHCMU cohort, all patients underwent preoperative breast DCE-MRI examination using a 3.0 T GE system with specialized breast coils. The imaging was performed using a T1-weighted sequence (adopting 3D DISCO technology)^[1]^. The imaging parameters were as follows: repetition time (TR) = 4.9 ms, echo time (TE) = 1.7 ms, field of view (FOV) = 360 mm × 360 mm, matrix size = 256 × 256, slice thickness = 1.4 mm, slice gap = 1.4 mm, flip angle = 15°, and number of slices = 120. After acquiring a precontrast phase of the T1CE sequence, a contrast agent (gadodiamide, GE Healthcare, Ireland) was injected intravenously at a dosage of 0.1 mmol/kg at a rate of 2 mL/sec, followed by a 20 mL saline flush.

Vision Transformer Model Architecture

The Vision Transformer (ViT) is a deep learning architecture that leverages the multi-head self-attention mechanism to model long-range dependencies within an image^[2]^. Unlike Convolutional Neural Network (CNN), which relies on localized receptive fields to extract hierarchical features, ViT operates on image patches and processes global contextual information. This characteristic makes ViT particularly advantageous for medical image analysis, where subtle and spatially dispersed features may be crucial for diagnosis. By capturing both local and global relationships within medical images, ViT provides a complementary approach to CNNs, which are effective in local feature extraction but may be less suited for capturing long-range dependencies between image regions. The self-attention mechanism in ViT enables information from all patches to be processed collectively, making it more suitable for analyzing complex spatial patterns in medical imaging. The ViT architecture is composed of the following fundamental modules:

- Patch Embedding: In the ViT architecture, the input image is first divided into fixed-size, non-overlapping patches (e.g., 16×16 pixels). These patches are then linearly embedded into vectors of fixed dimensions, which serve as the input to the Transformer model. The patch embedding step replaces traditional convolutional operations and transforms the image into a sequence of patch tokens, making the model more flexible in handling different types of images.
- Position Embedding: Since ViT processes image patches as a sequence, it does not inherently preserve the spatial relationships between patches. To address this, position embeddings are added to the patch embeddings. These positional encodings provide the model with information about the relative or absolute position of each patch within the image, enabling it to retain spatial information despite the lack of a convolutional structure. This allows ViT to learn and maintain the spatial context when processing the image.
- Transformer Encoder: The core of ViT is the Transformer encoder, composed of multiple stacked layers. Each layer includes two main components: (a) multi-head self-attention layers and (b) Multilayer Perceptron (MLP) blocks. The multi-head self-attention mechanism enables the model to compute relationships between all patches in the image, learning long-range dependencies and capturing global context. Multiple attention heads allow the model to focus on different aspects of the input data simultaneously, which improves its ability to learn diverse features from the image. Following the multi-head self-attention layers, each Transformer layer applies an MLP block. Each token is processed independently through two fully connected layers with a Gaussian Error Linear Unit (GELU) activation function in between. The Transformer encoder operates in a layered manner, with each layer refining the image representation by sequentially applying multi-head self-attention followed by the MLP block. This process enables ViT to effectively learn both local and global dependencies within the image.

By leveraging these architectural components, ViT provides a scalable and adaptable approach to capture complex spatial and hierarchical patterns, making the model highly effective for image classification tasks.

Model Training and Evaluation Details

- Data Preprocessing. The input data for model training consisted of early-phase dynamic contrast-enhanced MRI (DCE-MRI) images. Tumor regions were identified using manually annotated segmentation masks, and regions of interest (ROIs) were extracted with a 10-pixel margin surrounding the tumor boundaries to capture peritumoral features. The following preprocessing steps were applied to ensure consistency and enhance image quality: (a) Intensity Normalization. Intensity values were normalized using Z-score normalization to standardize pixel intensity distributions. (b) Contrast Enhancement: Contrast Limited Adaptive Histogram Equalization (CLAHE) was used to improve local contrast in the images. (c) Soft Mask Generation: Segmentation masks were smoothed with Gaussian blurring to create soft masks, which were then blended with the original images. (d) Resizing: The extracted ROIs were resized to 224 × 224 pixels to match the input dimensions of the Vision Transformer (ViT).
- Data Augmentation. To improve model robustness and generalizability, data augmentation techniques were applied to the training data: (a) Random Rotations: Up to ± 15°. (b) Random Flips. Horizontal and vertical. (c) Random Resized Cropping. Scale range of 0.9–1.1 and aspect ratio range of 0.9–1.1. (d) Gaussian Noise Addition. Mean = 0, standard deviation = 0.01.
- Model Development. Optimization of the model was performed using the Adam optimizer (learning rate = 1 × 10^-4^, weight decay = 1 × 10^-4^) with a cosine annealing learning rate scheduler. The learning rate $\boldsymbol{\eta}_{\boldsymbol{t}}$ was adjusted at each epoch $\boldsymbol{t}$ according to the following formula:

$\boldsymbol{\eta}_{\boldsymbol{t}}\boldsymbol{=}\boldsymbol{\eta}_{\text{min}}\boldsymbol{+}\frac{\boldsymbol{1}}{\boldsymbol{2}}\left( \boldsymbol{\eta}_{\text{max}}\boldsymbol{-}\boldsymbol{\eta}_{\text{min}} \right)\left( \boldsymbol{1}\boldsymbol{+}\cos\left( \frac{\boldsymbol{t}}{\boldsymbol{T}_{\text{max}}}\boldsymbol{\pi} \right) \right)$,

where $\boldsymbol{\eta}_{\text{max}}$ = 1 × 10^-4^, $\boldsymbol{\eta}_{\text{min}}$ = 1 × 10^-6^, and $\boldsymbol{T}_{\text{max}}$ = 10 epochs. This scheduler facilitates a gradual reduction in the learning rate, improving convergence during training.

The binary cross-entropy loss function with logits was defined as:

$\text{BCE}\left( \boldsymbol{p,y} \right)\boldsymbol{=-}\frac{\boldsymbol{1}}{\boldsymbol{N}}\sum_{\boldsymbol{i=1}}^{\boldsymbol{N}} \left[ \boldsymbol{y}_{\boldsymbol{i}}\log\boldsymbol{\sigma}\left( \boldsymbol{p}_{\boldsymbol{i}} \right)\boldsymbol{+}\left( \boldsymbol{1-}\boldsymbol{y}_{\boldsymbol{i}} \right)\log\left( \boldsymbol{1-\sigma}\left( \boldsymbol{p}_{\boldsymbol{i}} \right) \right) \right]$,

where $\boldsymbol{p}_{\boldsymbol{i}}$ is the predicted logit for sample $\boldsymbol{i}$, $\boldsymbol{y}_{\boldsymbol{i}}\boldsymbol{\in}\boldsymbol{\{}\boldsymbol{0,1}\boldsymbol{\}}$ is the ground truth label, and $\boldsymbol{\sigma}\left( \boldsymbol{p}_{\boldsymbol{i}} \right)$ is the sigmoid activation function:

$$\boldsymbol{\sigma}\left( \boldsymbol{p}_{\boldsymbol{i}} \right)\boldsymbol{=}\frac{\boldsymbol{1}}{\boldsymbol{1+}\boldsymbol{e}^{\boldsymbol{-}\boldsymbol{p}_{\boldsymbol{i}}}}$$

This loss function combines sigmoid activation and binary cross-entropy to ensure numerical stability and robust optimization for binary classification tasks.

- Training and Early Stopping. The training process included a batch size of 16 and a maximum of 50 epochs. An early stopping mechanism was employed with a patience of 8 epochs, saving the model checkpoint with the lowest validation loss.
- Implementation Environment. The models were implemented using PyTorch (https://pytorch.org) with support from SimpleITK for medical image processing (https://simpleitk.readthedocs.io). All experiments were conducted on an NVIDIA GPU using CUDA (https://developer.nvidia.com/cuda-zone) for computational acceleration.

Sample processing for total RNA extraction

For quality control, fresh frozen tumor tissues were macrodissected, and samples containing more than 50% stromal tissue were filtered out. Total RNA was purified from tissues previously stored in RNAlater solution using the miRNeasy Mini Kit (Qiagen, #217004) according to the manufacturer's instructions. RNA integrity was assessed using an Agilent 4200 Bioanalyzer with RNA ScreenTape (Agilent Inc.), and RNA concentrations were determined using a NanoDrop ND-8000 spectrophotometer (Thermo Fisher Scientific Inc.). The purity and quantity of RNA were further estimated by measuring the absorbance at 260 nm (A260) and 280 nm (A280) using a NanoDrop 2000 spectrophotometer (Thermo Scientific, Wilmington, DE, USA). Extracted RNA was considered pure and suitable for future experiments when the A260/A280 ratio was within the range of 1.8-2.1.

RNA sequencing procedures

RNA libraries were constructed using a ribosomal RNA depletion method. Ribosomal RNA was removed with the Ribo-off rRNA Depletion Kit (H/M/R) (Vazyme #N406, Vazyme Biotech Co., Ltd., Nanjing, China), and RNA libraries were generated using the VAHTS Universal V8 RNA-seq Library Prep Kit for Illumina (Vazyme #NR605, Vazyme Biotech Co., Ltd., Nanjing, China). Specifically, fragmented RNA was reverse-transcribed into cDNA, followed by 3' poly(A) tail modification. Next, adapters were ligated to the cDNA, and PCR amplification was performed to enrich the libraries. During quality control (QC), Qubit 4.0 (Thermo Fisher Scientific Inc.) was used to measure library concentration, and the Agilent 2200 Bioanalyzer (Agilent Inc.) was used to assess fragment size distribution. Libraries were sequenced on the Illumina NovaSeq platform using paired-end reads of 150 bp.

Raw Illumina sequence data were demultiplexed and converted to FASTQ format, with adapter and low-quality sequences being trimmed. Sample reads were aligned to the hg38 human genome reference using HISAT2. Gene expression was quantified by calculating fragments per kilobase of transcript per million mapped reads (FPKM) using StringTie and Ballgown. To ensure accuracy in gene expression measurements, genes with FPKM values of 0 in more than 30% of samples were excluded from subsequent analyses.

Bioinformatic operations and analysis

Differentially expressed genes (DEGs) were identified using the limma package in R, with the Benjamini-Hochberg (BH) correction for multiple testing. DEGs were defined by an adjusted p-value < 0.05 and |log_2_FC| > 0.3. Genes were classified as increased if log_2_FC > 0.3 and decreased if log_2_FC < −0.3. Genes not meeting these criteria were labeled as Not Significant.

Gene Ontology (GO) enrichment analysis was performed using the clusterProfiler package with the org.Hs.eg.db annotation database.^[3]^ Three ontologies—Biological Process (BP), Cellular Component (CC), and Molecular Function (MF)—were assessed with significance determined by p-value < 0.05 and q-value < 0.05. The results were summarized and exported for further analysis. KEGG pathway enrichment analysis was conducted using the enrichKEGG function from the clusterProfiler package.^[4]^ Only genes with Entrez IDs corresponding to the upregulated and downregulated DEGs were included. The significance threshold was set to p-value < 0.05 and q-value < 0.05.

In addition to GO and KEGG pathway enrichment analysis, we performed Gene Set Enrichment Analysis (GSEA) on the differentially expressed genes (DEGs) to further explore the functional annotations and pathways associated with the identified gene expression patterns.^[5]^ Using the preranked gene list based on the log2 fold changes of the DEGs, we conducted GSEA using both Gene Ontology (GO) and KEGG pathway databases. All statistical analyses were conducted in R.

Supplementary Tables

Table S1: MRI Scanner and Scan Parameters

|  | Field strength | Brand | TR  (msec) | TE  (msec) | FOV  (mm²) | Slice  thickness  (mm) | Slice gap  (mm) | Matrix | Number of slices |
| --- | --- | --- | --- | --- | --- | --- | --- | --- | --- |
| FUSCC cohort | 1.5T | Aurora | 29 | 4.8 | 360×360 | 1.5 | 1.5 | 360×360 | 108 |
| GFPH cohort | 1.5T | United  Imaging | 5.1 | 2.1 | 360×360 | 1.2 | 1.2 | 416×416 | 120 |
| FHCMU cohort | 3.0T | GE | 4.9 | 1.7 | 360×360 | 1.4 | 1.4 | 256×256 | 120 |

Note.—GE = General Electric, TR = time of repetition, TE = time of echo, FOV = field of view.

Table S2: Patient Characteristics in the Training, Validation, and External Test Sets for Distinguishing HER2-Zero from HER2-Low/Positive

| Variable | Training set  (n = 567) | Validation set  (n = 141) | *P* Value | Test set 1  (n = 80) | Test set 2  (n = 101) |
| --- | --- | --- | --- | --- | --- |
| Age (y)* | 52.8 (10.4) | 51.9 (11.3) | .39 | 54.4 (9.3) | 49.2 (10.4) |
| Menopause  YES  NO  NA | 323 (57.0)  240 (42.3)  4 (0.7) | 80 (56.7)  61 (43.3)  0 | .97 | 56 (70.0)  24 (30.0)  0 | 44 (43.6)  57 (56.4)  0 |
| Histology |  |  | .23 |  |  |
| Invasive | 523 (92.2) | 125 (88.7) |  | 73 (91.3) | 93 (92.1) |
| Others | 44 (0.8) | 16 (11.3) |  | 7 (8.7) | 8 (7.9) |
| T stage |  |  | .56 |  |  |
| 1 | 241 (42.5) | 62 (44.0) |  | 15 (18.8) | 6 (5.9) |
| 2 | 308 (54.3) | 71 (50.4) |  | 15 (18.8) | 64 (63.4) |
| 3 | 14 (2.5) | 5 (3.5) |  | 1 (1.3) | 21 (20.8) |
| 4 | 0 | 0 |  | 0 | 10 (9.9) |
| NA | 4 (0.7) | 3 (2.1) |  | 49 (61.1) | 0 |
| N stage |  |  | .67 |  |  |
| 0 | 292 (51.5) | 73 (51.8) |  | 23 (28.6) | 17 (16.8) |
| 1 | 149 (26.3) | 33 (23.4) |  | 6 (7.5) | 57 (56.4) |
| 2 | 68 (12.0) | 21 (14.9) |  | 1 (1.3) | 15 (14.9) |
| 3 | 55 (9.7) | 11 (7.8) |  | 1 (1.3) | 12 (11.9) |
| NA | 3 (0.5) | 3 (2.1) |  | 49 (61.1) | 0 |
| Grade |  |  | .87 |  |  |
| 1 | 4 (0.7) | 1 (0.7) |  | 8 (10.0) | 0 |
| 2 | 225 (39.7) | 47 (33.3) |  | 35 (43.7) | 73 (72.3) |
| 3 | 300 (52.9) | 69 (49.0) |  | 12 (15.0) | 27 (26.7) |
| NA | 38 (6.7) | 24 (17.0) |  | 25 (31.3) | 1 (1.0) |
| ER status |  |  | .41 |  |  |
| Positive | 326 (57.5) | 75 (53.2) |  | 57 (71.2) | 64 (63.4) |
| Negative | 241 (42.5) | 66 (46.8) |  | 23 (28.8) | 37 (36.6) |
| PR status |  |  | .20 |  |  |
| Positive | 282 (49.7) | 61 (43.3) |  | 51 (61.7) | 66 (65.3) |
| Negative | 285 (50.3) | 80 (56.7) |  | 29 (36.3) | 35 (34.7) |
| HER2 status |  |  | .63 |  |  |
| Positive/Low | 459 (81.0) | 111 (78.7) |  | 70 (87.5) | 75 (74.3) |
| Zero | 108 (19.0) | 30 (21.3) |  | 10 (12.5) | 26 (25.7) |

Note.—Except where indicated, data are numbers of women with percentages in parentheses. HER2 = human epidermal growth factor receptor 2; ER = estrogen receptor; PR = progesterone receptor; NA = not available.

* Data are means ± SDs.

Table S3: Patient Characteristics in the Training, Validation, and External Test Sets for Distinguishing HER2-Low from HER2-Positive

| Variable | Training set  (n = 456) | Validation set  (n = 114) | *P* Value | Test set 1  (n = 70) | Test set 2  (n = 75) |
| --- | --- | --- | --- | --- | --- |
| Age (y)* | 52.6 (10.4) | 51.7 (10.1) | .75 | 54.8 (9.1) | 48.5 (10.2) |
| Menopause  YES  NO  NA | 261 (57.3)  193 (42.3)  2 (0.4) | 60 (52.6)  54 (47.4)  0 | .41 | 49 (70.0)  21 (30.0)  0 | 30 (40.0)  45 (60.0)  0 |
| Histology |  |  | .91 |  |  |
| Invasive | 420 (92.1) | 104 (91.2) |  | 65 (92.9) | 70 (93.3) |
| Others | 36 (7.9) | 10 (8.8) |  | 5 (7.1) | 5 (6.7) |
| T stage |  |  | .63 |  |  |
| 1 | 191 (41.9) | 44 (38.5) |  | 13 (18.6) | 4 (5.3) |
| 2 | 247 (54.2) | 66 (57.9) |  | 13 (18.6) | 49 (65.3) |
| 3 | 15 (3.3) | 2 (1.8) |  | 1 (1.4) | 14 (18.7) |
| 4 | 0 | 0 |  | 0 | 8 (10.7) |
| NA | 3 (0.6) | 2 (1.8) |  | 43 (61.4) | 0 |
| N stage |  |  | .71 |  |  |
| 0 | 226 (49.6) | 63 (55.3) |  | 19 (27.1) | 10 (13.3) |
| 1 | 118 (25.9) | 27 (23.7) |  | 6 (8.6) | 45 (60.1) |
| 2 | 61 (13.4) | 15 (13.2) |  | 1 (1.4) | 10 (13.3) |
| 3 | 48 (10.5) | 9 (7.8) |  | 1 (1.4) | 10 (13.3) |
| NA | 3 (0.6) | 0 |  | 43 (61.5) | 0 |
| Grade |  |  | .89 |  |  |
| 1 | 4 (0.8) | 1 (0.9) |  | 8 (11.4) | 0 |
| 2 | 185 (40.6) | 43 (37.7) |  | 30 (42.9) | 58 (77.4) |
| 3 | 231 (50.7) | 59 (51.8) |  | 12 (17.1) | 16 (21.3) |
| NA | 36 (7.9) | 11 (9.6) |  | 20 (28.6) | 1 (1.3) |
| ER status |  |  | .72 |  |  |
| Positive | 269 (59.0) | 70 (61.4) |  | 49 (70.0) | 52 (69.3) |
| Negative | 187 (41.0) | 44 (38.6) |  | 21 (30.0) | 23 (30.7) |
| PR status |  |  | .69 |  |  |
| Positive | 228 (50.0) | 60 (52.6) |  | 43 (61.4) | 55 (73.3) |
| Negative | 228 (50.0) | 54 (47.4) |  | 27 (38.6) | 20 (26.7) |
| HER2 status |  |  | .43 |  |  |
| Positive | 185 (40.6) | 41 (36.0) |  | 38 (54.3) | 22 (29.3) |
| Low | 271 (59.4) | 73 (64.0) |  | 32 (45.7) | 53 (70.7) |

Note.—Except where indicated, data are numbers of women with percentages in parentheses. HER2 = human epidermal growth factor receptor 2; ER = estrogen receptor; PR = progesterone receptor; NA = not available.

* Data are means ± SDs.

Supplementary Materials References:

[1] M. Saranathan, D. W. Rettmann, B. A. Hargreaves, S. E. Clarke, S. S. Vasanawala, *J Magn Reson Imaging* 2012, *35*, 1484.

[2] A. Dosovitskiy, L. Beyer, A. Kolesnikov, D. Weissenborn, X. Zhai, T. Unterthiner, M. Dehghani, M. Minderer, G. Heigold, S. Gelly, J. Uszkoreit, N. Houlsby, *An Image is Worth 16x16 Words: Transformers for Image Recognition at Scale*, arXiv 2021.

[3] T. Wu, E. Hu, S. Xu, M. Chen, P. Guo, Z. Dai, T. Feng, L. Zhou, W. Tang, L. Zhan, X. Fu, S. Liu, X. Bo, G. Yu, *Innovation (Camb)* 2021, *2*, 100141.

[4] K. M, G. S, *Nucleic acids research* 2000, *28*.

[5] A. Subramanian, P. Tamayo, V. K. Mootha, S. Mukherjee, B. L. Ebert, M. A. Gillette, A. Paulovich, S. L. Pomeroy, T. R. Golub, E. S. Lander, J. P. Mesirov, *Proc Natl Acad Sci U S A* 2005, *102*, 15545.

Supplemental figure legends


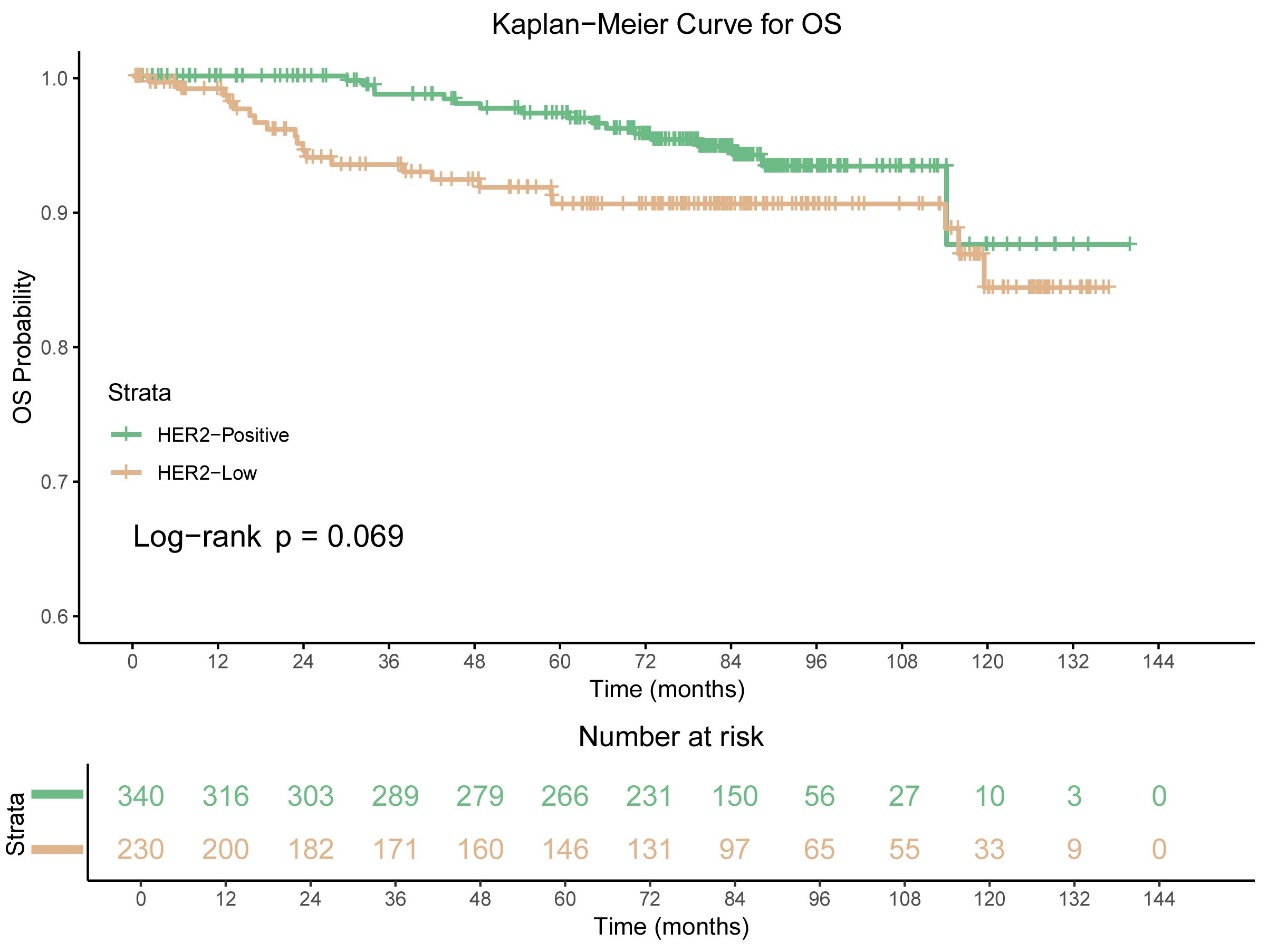


Figure S1. Kaplan–Meier analysis for overall survival (OS) based on actual HER2-low and HER2-positive groups (p = 0.069 by log-rank test) in FUSCC cohort.


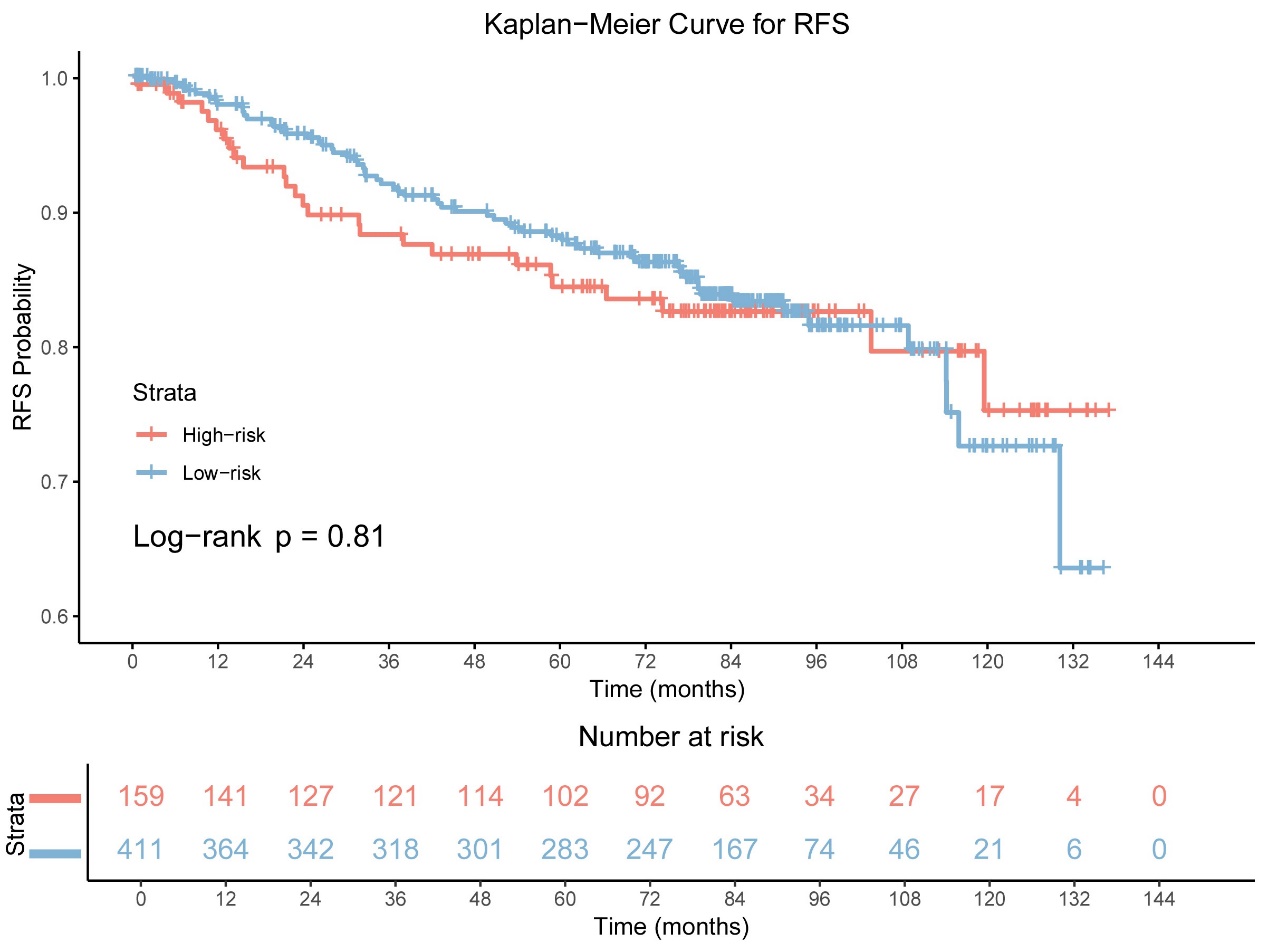


Figure S2. Kaplan–Meier analysis for relapse-free survival (RFS) based on high- and low-risk groups (cutoff = 0.5) predicted by the Vision Transformer (ViT) model in distinguishing HER2-low from HER2-positive patients (p = 0.81 by log-rank test) in FUSCC cohort.


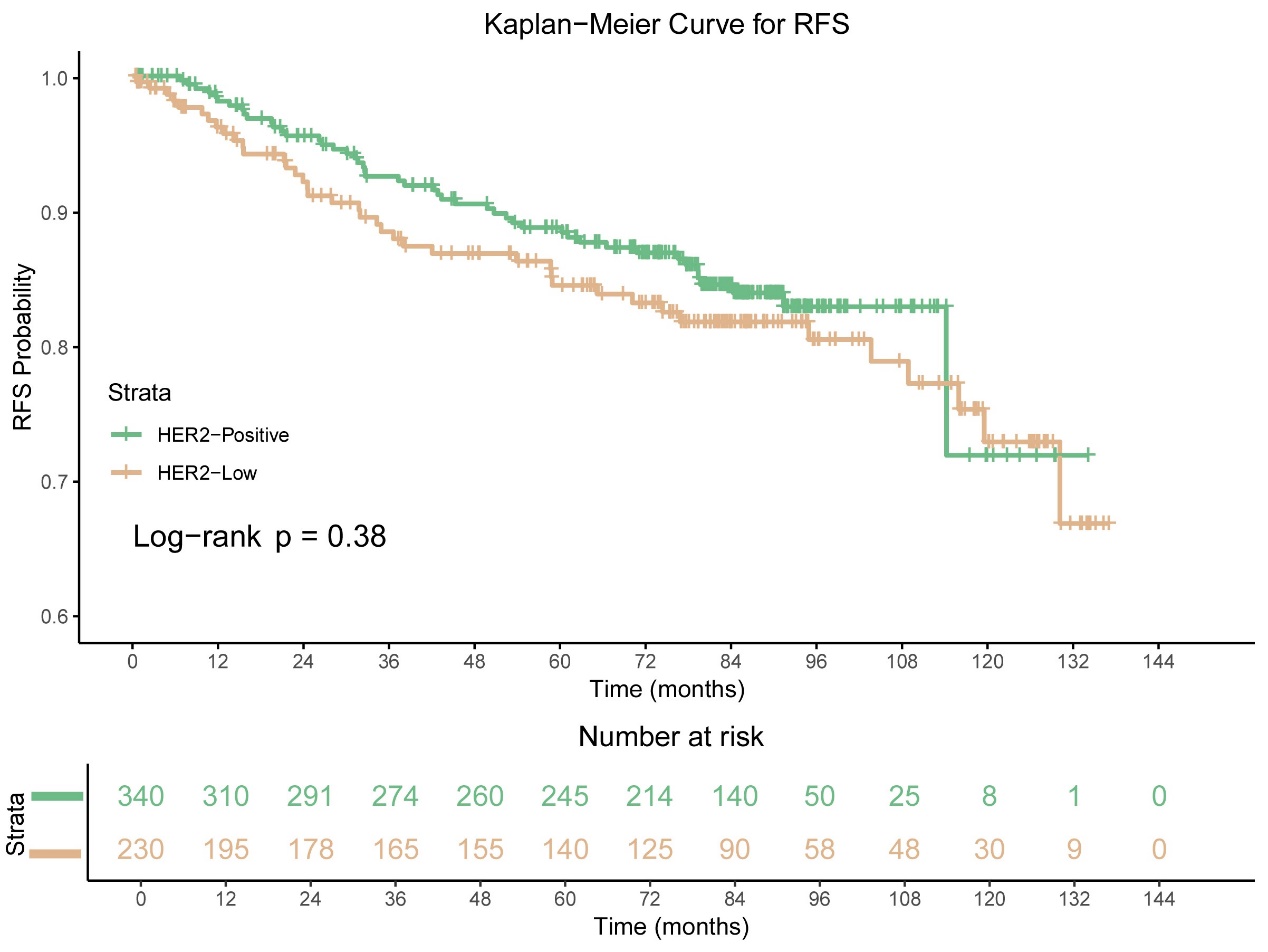


Figure S3. Kaplan–Meier analysis for relapse-free survival (RFS) based on actual HER2-low and HER2-positive groups (p = 0.38 by log-rank test) in FUSCC cohort.


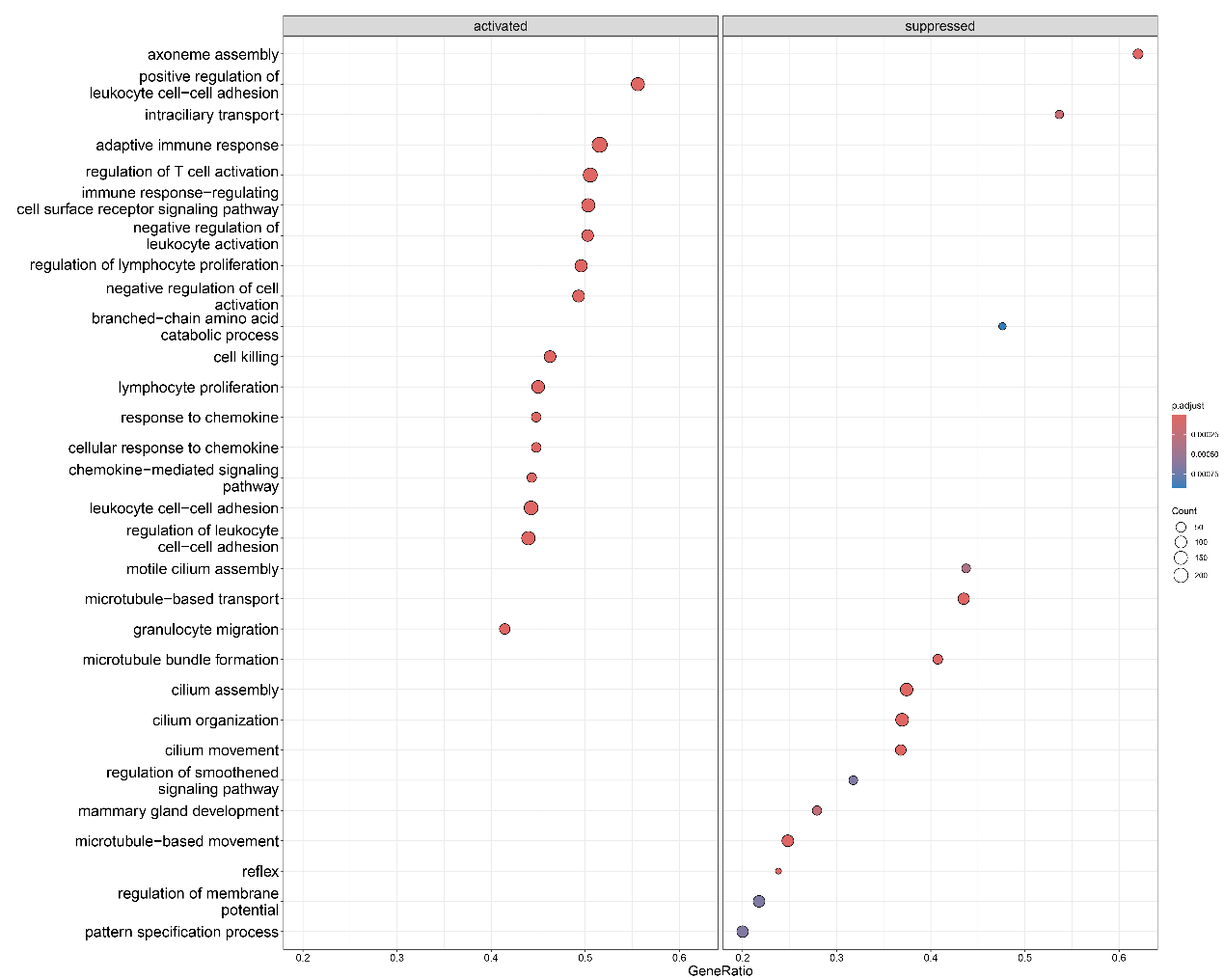


Figure S4. Gene Ontology biological process enrichment analysis of differentially expressed genes in predicted HER2-Positive and HER2-Low groups.


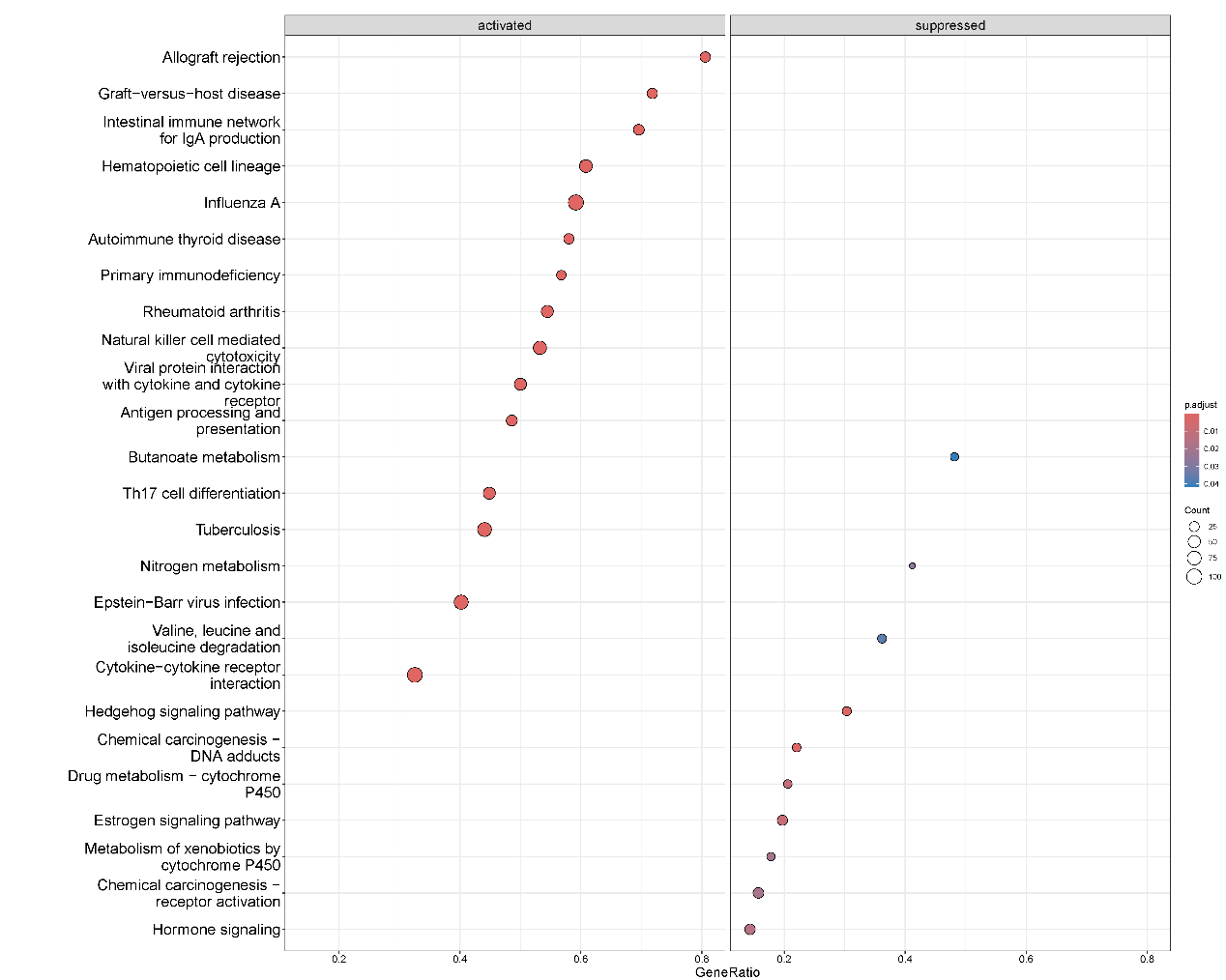


Figure S5. Kyoto Encyclopedia of Genes and Genomes enrichment analysis of differentially expressed genes in predicted HER2-Positive and HER2-Low groups.
